# Centromeres Convert but Don't Cross

**DOI:** 10.1371/journal.pbio.1000326

**Published:** 2010-03-09

**Authors:** Paul B. Talbert, Steven Henikoff

**Affiliations:** Howard Hughes Medical Institute, Fred Hutchinson Cancer Research Center, Seattle, Washington, United States of America

## Abstract

Geneticists have long known that centromeres suppress crossing over, but considerable evidence indicates that they appear to recombine. Confirmation of gene conversion in maize centromeres explains this paradox.

A long-standing problem in chromosome biology concerns the dynamic nature of centromeres. These chromosomal sites assemble the protein machines called kinetochores that connect chromosomes to the spindle microtubules for segregation to daughter cells during mitosis and meiosis. In multicelluar eukaryotes, centromeres are typically composed of highly homogeneous tandem repeats that evolve rapidly despite their highly conserved function [1]. For tandem repeats to evolve, a mutation must spread by some recombinational process, but a persistent dogma is that centromeres do not undergo homologous chromosome recombination (the shuffling of genetic segments between chromosomal pairs). New evidence [2] challenges this dogma and addresses the problem of rapidly evolving centromeres.

## The Role of Crossing Over in Meiosis

**Figure 1:**
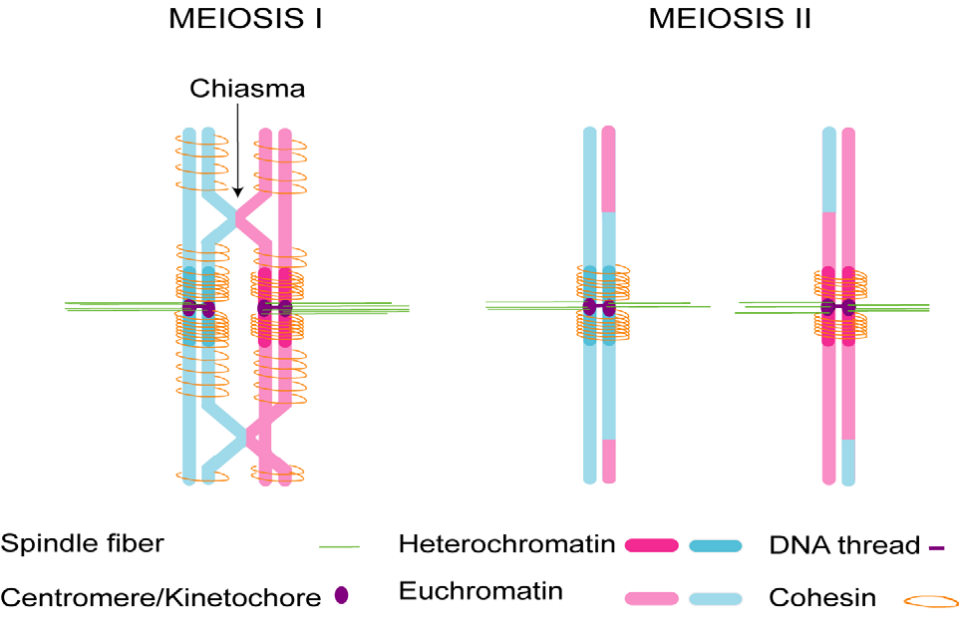
Chromosome connections in meiosis. Kinetochores attach homologous chromosomes to opposite halves of the spindle. Homologs are held together by chiasmata, in which recombinant chromatids cross each other. Sisters are held together by cohesins and possibly by catenation of centromeric DNA threads, which have been observed in human mitosis. Cohesion is released in two steps: on chromosome arms to resolve chiasmata and separate homologs in the first meiotic division; and around centromeres to separate sisters in the second meiotic division.

Centromeres do not act alone in orchestrating chromosome segregation. In order for sister kinetochores to properly disjoin (separate) and segregate chromosomes equally to daughter cells in mitosis, their sister chromatids must be linked so that the pulling forces from the two halves of the spindle generate tension to correctly orient the kinetochores, stabilize kinetochore attachments, and signal that kinetochores are ready to disjoin. Centromeres in multicellular eukaryotes are typically embedded in heterochromatin, the permanently condensed chromatin found around centromeres, in contrast to the euchromatic chromosome arms, which decondense between mitoses. Heterochromatin has been implicated in facilitating cohesion of sister chromatids around the centromere. This cohesion is mediated by cohesins, proteins that link the sisters together and that are enriched around centromeres [3], and possibly also by catenation (interlocking) of DNA threads observed between sister centromeres [4]. In most eukaryotes, homologs become physically linked during meiosis through the recombinational process of “crossing over”—the breakage and reciprocal reunion of homologous chromatids, resulting in a chiasma, the point where recombinant chromatids cross over each other (Figure 1). Failure to cross over is a major source of non-disjunction (improper segregation) at the first meiotic division in animals [5],[6], underscoring the importance of chiasmata for segregation of homologs.

As early as 1930, observations on the distribution of chiasmata along chromosomes led Karl Sax to predict that crossing over (and hence genetic recombination) is reduced around the centromere [7], and this “centromere effect” was verified in the fruitfly *Drosophila melanogaster* soon afterward [8]. Suppression of crossing over around or in centromeres has since been verified in several animals [9],[10], plants [11]–[14], and fungi [15],[16], with estimates of crossover suppression ranging from 5-fold to >200-fold in different organisms.

Why is crossing over suppressed around centromeres? In *Drosophila* [5], humans [6], and budding yeast (*Saccharomyces cerevisiae*) [17], non-disjunction events at the second meiotic division are enriched in centromere-proximal crossovers. This suggests that crossovers that are too close to the centromere disrupt pericentric sister chromatid cohesion, leading to premature separation of sister chromatids, which then segregate randomly. Thus, selective pressure to reduce crossing over near the centromere is likely to be strong. Crossing over within the centromere itself could be even more deleterious, leading to attachment of the centromere to both halves of the spindle, resulting in chromosome breakage and loss.

## Centromeres, Heterochromatin, and Crossover Suppression

How is crossing over suppressed at centromeres? The location of centromeres in heterochromatin raises the possibility that the crossover suppression seen at the centromere may simply be a property of the surrounding heterochromatin. Early attempts to separate heterochromatin from the centromere utilized inversions of pericentric heterochromatin on the *Drosophila* X chromosome and suggested that the centromere can suppress recombination independently of its flanking heterochromatin [18]. Subsequent work confirmed that heterochromatin also suppresses crossing over [19], consistent with its proposed role in facilitating cohesion. An increase in crossovers in *Drosophila* mutants that affect heterochromatin structure support the role of heterochromatin in suppressing pericentric crossovers [20]. Crossover suppression in plants also appears to be a feature of both centromeres and flanking heterochromatin. In *Arabidopsis thaliana*, crossing over is reduced >200-fold in the 2.3-Mb centromere region of Chromosome I, and 10–50 fold by the 1-Mb heterochromatic flanking regions [12],

At the molecular level, centromeres are distinguished from both heterochromatin and euchromatin by specialized nucleosomes containing the centromere-specific histone H3 variant known as CENP-A or CenH3, which is necessary to form the kinetochore. Occasionally functional CenH3-containing centromeres can arise on DNA that was previously non-centromeric and be faithfully transmitted (neocentromeres), indicating that centromere inheritance is epigenetic, dependent on the presence of CenH3 nucleosomes, not on specific DNA sequences (reviewed in [1]). Despite the apparent irrelevance of centromeric DNA sequence to kinetochore function, natural centromeres in plants and animals are usually composed of Mb-sized tandem arrays of short (150–180 bp) noncoding “satellite” repeats. These arrays may also be rich in transposon insertions, probably because suppression of crossing over prevents their elimination through recombination. The same or similar repeats comprise the flanking pericentric heterochromatin, underscoring the epigenetic specification of centromeres by CenH3 nucleosomes.

Although both centromeres and pericentric heterochromatin are rich in repetitive elements, repeats per se do not appear to be necessary for crossover suppression. For example, centromere 8 of rice (*Oryza sativa*), which has only very little satellite DNA, lacks detectable crossovers in a 2.3-Mb span around the 750-kb centromere region that contains discontinuous blocks of CenH3-containing nucleosomes. Remarkably, there is little difference in gene activity, transposon composition, or abundance of common histone modifications between this recombination-free region and adjacent recombining regions [21], suggesting that crossover suppression does not depend on DNA sequence but instead is epigenetic.

A clearer separation of centromere and heterochromatin effects can be found in budding yeast, which is unusual in having “point” centromeres that are only ∼120 bp in length [22], harbor a single CenH3 nucleosome [23] and lack surrounding heterochromatin. Suppression of crossing over at yeast centromeres is modest, estimated at only 3–6 fold, and extends over only about 10 kb or less [15],[24], although this represents as much as 80 times the length of the centromere itself. This suppression is eliminated by a point mutation in the centromere that renders it unable to assemble a functional kinetochore [25], strongly suggesting that the kinetochore mediates suppression.

## Satellite Arrays and Recombination

**Figure 2:**
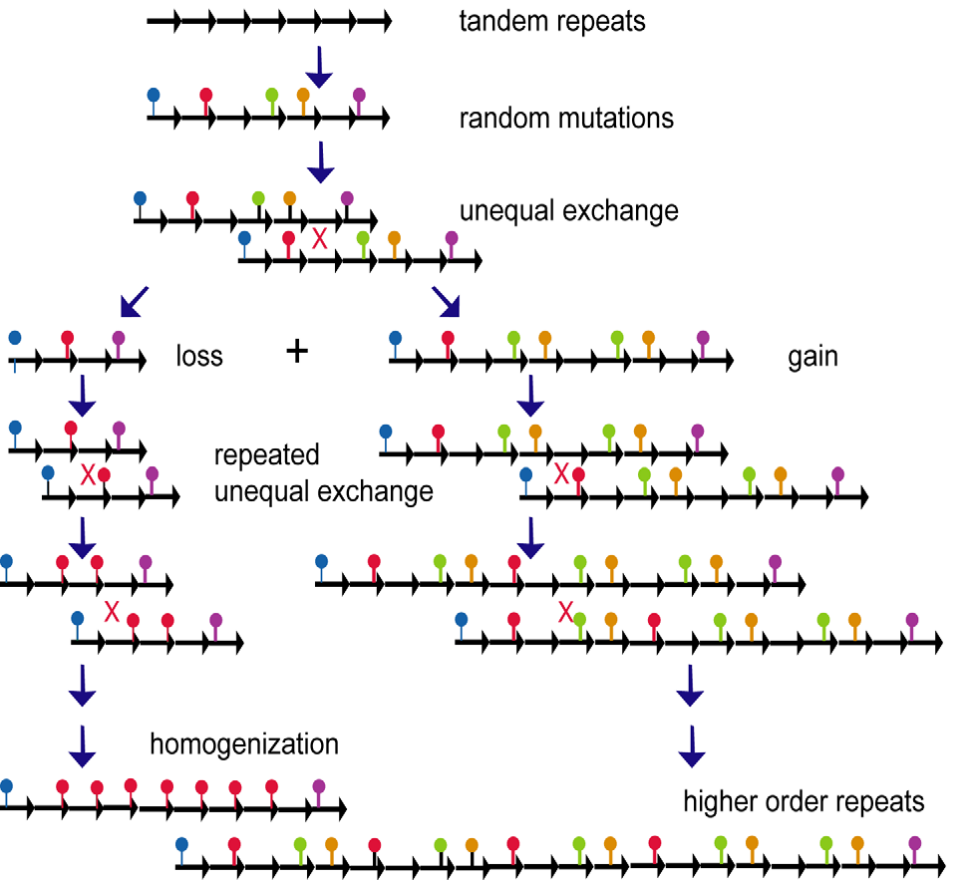
Unequal exchange in satellite arrays. Identical tandem satellite repeats become diversified over time by mutation. Unequal exchange results in gain or loss of tandem repeats. Repeated exchange can lead to homogenization of satellite repeats (left). If the unit of exchange consists of multiple diverged monomers, higher-order repeats are generated (right).

Although crossing over is suppressed around centromeres, the tandem satellite array structure that is typical for most centromeres is best explained by extensive and repeated recombination. The generation of such arrays has been modeled as a recombinational process of random unequal exchange [26]. Unequal exchange can act on variation in the individual satellite monomers due to mutation to lead to expansion of new repeat variants and/or formation of higher-order repeats (Figure 2), as well as eliminating variation in monomers (homogenization). In the human X chromosome, the CenH3-containing chromatin is found centrally in the most recent and most homogeneous higher-order repeats of the human alpha satellite array, whereas the older and more diversified satellite monomers comprise the flanking pericentric heterochromatin [27]. Analysis of the CentO satellites in centromeres of rice revealed segmental duplications, insertions and deletions, inversions, and reshuffling of variant satellite monomers [28]. Unequal exchange occurs at a high frequency between sister centromeres in mitotically cycling mouse (*Mus musculus*) chromosomes and is negatively regulated by DNA methylation, without which loss of repeats occurs [29]. However, it is unknown whether these recombination events can be transmitted through meiosis to the next generation. These observations provide evidence of extensive recombination in centromeres over evolutionary time scales and underscore the instability of repeat arrays to recombination and the necessity of suppressing crossing over in order to maintain centromere structure. How can this evidence for recombination in centromeres be reconciled with crossover suppression?

## Conversion in Centromeres

**Figure 3:**
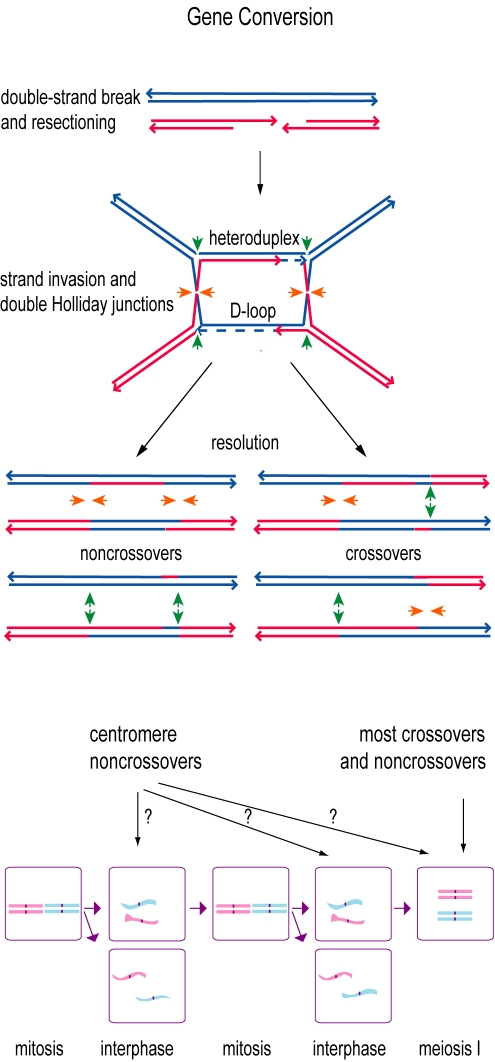
Gene conversion. In a popular model for gene conversion [41], recombination begins with a double-strand break in one chromosome (red) and resectioning (chewing back) of the 5′ ends of the break. A free 3′ end invades the homolog (blue) forming a D-loop and heteroduplex DNA. Non-reciprocal DNA synthesis fills in missing DNA (dashed arrows), forming two Holliday junctions, which may be resolved as either crossovers or noncrossovers, depending on which strands are cut (green and orange arrows). Gene conversion between homologs takes place in meiosis (bottom right), generating both crossovers and noncrossovers. Centromeres might undergo noncrossover conversion in mitotically cycling cells during growth and development (bottom left and center) as part of double-strand break repair. Conversion between homologs would be necessary to repair breaks prior to replication, when there is no cohering sister centromere to use as a repair template.

In the same year that Sax predicted the centromere effect on crossing over, a new model of recombination, called gene conversion, was proposed to explain non-reciprocal recombination events in mosses and basidiomycetes [30]. Gene conversion is now thought to be a normal part of the homologous recombination pathway in which a programmed double-strand break in the DNA is repaired by copying a short (usually ∼2 kb or less) stretch of the homologous chromosome. The resulting conversion event may then be resolved into a either a crossover or a noncrossover (Figure 3). Could noncrossover gene conversions contribute to recombination in centromeres in the absence of crossing over?

The localized nature of gene conversion makes it significantly more difficult to detect than crossing over. A key problem is the need for numerous closely spaced unique markers in the highly repetitive sequences of the centromere and pericentromere. Consequently this question has been most thoroughly addressed in budding yeast, which lacks centromeric and pericentric repeats. Most studies have concluded that gene conversion is moderately suppressed (4- to 7-fold) at yeast centromeres, along with crossing over [24],[25]. However, initiating double-strand breaks are not found within the point centromeres, but rather nearby [31],[32]. One study reported that when nearby conversion events were examined, the conversion tract frequently included part or all of the centromere, and concluded that conversion rates at centromeres were not different than in non-centromeric regions [33]. Thus, the small size of yeast centromeres means that the relationship between the kinetochore and suppression of gene conversion has remained ambiguous.

To determine whether gene conversion events can occur within large centromeres and provide the recombination events underlying both satellite homogenization and centromere diversity, a new report by Shi et al. [2] studied events within the centromeres of maize (*Zea mays*). They developed 238 centromeric markers based on insertion polymorphisms of the centromere-specific transposon CRM2 that map to all ten maize centromeres. To verify their centromeric location, centromeric chromatin was immunoprecipitated with an anti-CenH3 antibody. CenH3 is distributed discontinuously in maize centromeres [34] and only about 30% of CRM sequences can be immunoprecipitated with anti-CenH3 [34]–[36]. Markers were then assessed in two parental lines and in 94 recombinant inbred lines derived from their progeny. As expected, no crossovers were observed. However, in two cases a single marker from one parent was gained in a centromere with all markers of the other parent, indicating a conversion event. The formal possibility that these events represent double crossovers is unlikely given the failure to find single crossovers.

Shi et al. then proceeded to assess their marker set in 53 highly diverse inbred lines representing the diversity of maize and found widespread evidence for marker recombination since the origin of maize, perhaps 9,000 years ago [37]. They could distinguish between crossovers and noncrossover conversions by determining the linkage disequilibrium (LD), or tendency of markers in a population to occur together on the same chromosome. In crossing over, LD decreases with distance, whereas the short conversion tracts of noncrossovers show no relationship between LD and distance, because the conversion of one marker ordinarily has no effect on the coinheritance of its neighbors. No correlation was found between distance and LD in centromere 2, which has been fully sequenced [36], consistent with noncrossover conversion. Two population genetic methods gave similar estimates of the conversion rate of >1×10^−5^ conversions per marker per generation, a rate not dissimilar to one estimate for the conversion rate on the chromosome arms [38].

These results are significant both for understanding the regulation of recombination in maize and for understanding the evolution of centromeres. Except in yeasts, studying recombination in centromeres has hitherto been largely a matter of inferring the occurrence of ancient events based on present-day sequences. The results of Shi et al. show that it is possible to study centromeric recombination in action in a multicellular eukaryote. They also confirm that such recombination can take place between homologs and not solely between sisters, with implications for the creation and spread of new centromere variants. Meiotic recombination involves complete end-to-end pairing of homologs, whereas a gene conversion event requires only a local homologous interaction, and it is possible that the observed conversion events occurred during mitotic development rather than during meiosis (Figure 3). For example, the mitotic threads seen to connect human sister centromeres [4] might sometimes be resolved via breakage events that initiate repair by homologous recombination. By this scenario, the surprisingly high level of genetic exchange observed by Shi et al. might be a consequence of the many mitoses that occur for each meiotic generation within a maize lineage.

Widespread gene conversion might be a general feature of centromeres of multicellular eukaryotes. Human centromeres are composed of higher-order alpha satellite repeat arrays [27], and evidence for their periodic homogenization suggests an underlying gene conversion mechanism [39]. As is the case for unequal exchange between sisters, which is the most attractive explanation for the large expansions and contractions of alpha satellite repeat arrays, centromeric gene conversion challenges the widely held perception of centromeres as genetically stable regions of the genome. The actions of gene conversion and unequal exchange provide variation that makes possible Darwinian competition of centromeres that may lead to their rapid diversification [40]. Thus the problem of both homogenization and diversification of centromeres in the absence of crossovers can be resolved.

## Abbreviations

CenH3: centromeric histone H3 variant
CRM2: centromere-specific retrotransposon of maize 2
LD: linkage disequilibrium

## References

[1] Malik H. S, Henikoff S (2009). Major evolutionary transitions in centromere complexity.. Cell.

[2] Shi J, Wolf S. E, Burke J. M, Presting G. G, Ross-Ibara J (2010). Widespread gene conversion in centromere cores.. PLoS Biol.

[3] Gartenberg M (2009). Heterochromatin and the cohesion of sister chromatids.. Chromosome Res.

[4] Wang L. H, Schwarzbraun T, Speicher M. R, Nigg E. A (2008). Persistence of DNA threads in human anaphase cells suggests late completion of sister chromatid decatenation.. Chromosoma.

[5] Koehler K. E, Boulton C. L, Collins H. E, French R. L, Herman K. C (1996). Spontaneous X chromosome MI and MII nondisjunction events in *Drosophila melanogaster* oocytes have different recombinational histories.. Nat Genet.

[6] Lamb N. E, Sherman S. L, Hassold T. J (2005). Effect of meiotic recombination on the production of aneuploid gametes in humans.. Cytogenet Genome Res.

[7] Sax K (1930). Chromosome structure and the mechanism of crossing over.. J Arnold Arb.

[8] Beadle G. W (1932). A possible influence of the spindle fibre on crossing-over in *Drosophila*.. Proc Natl Acad Sci U S A.

[9] Mahtani M. M, Willard H. F (1998). Physical and genetic mapping of the human X chromosome centromere: Repression of recombination.. Genome Res.

[10] Rahn M. I, Solari A. J (1986). Recombination nodules in the oocytes of the chicken, *Gallus domesticus*.. Cytogenet Cell Genet.

[11] Sherman J. D, Stack S. M (1995). Two-dimensional spreads of synaptonemal complexes from solanaceous plants. VI. high-resolution recombination nodule map for tomato (*Lycopersicon esculentum*).. Genetics.

[12] Haupt W, Fischer T. C, Winderl S, Fransz P, Torres-Ruiz R. A (2001). The centromere1 (CEN1) region of *Arabidopsis thaliana*: Architecture and functional impact of chromatin.. Plant J.

[13] Harushima Y, Yano M, Shomura A, Sato M, Shimano T (1998). A high-density rice genetic linkage map with 2275 markers using a single F2 population.. Genetics.

[14] Anderson L. K, Doyle G. G, Brigham B, Carter J, Hooker K. D (2003). High-resolution crossover maps for each bivalent of *Zea mays* using recombination nodules.. Genetics.

[15] Lambie E. J, Roeder G. S (1986). Repression of meiotic crossing over by a centromere (CEN3) in *Saccharomyces cerevisiae*.. Genetics.

[16] Nakaseko Y, Adachi Y, Funahashi S, Niwa O, Yanagida M (1986). Chromosome walking shows a highly homologous repetitive sequence present in all the centromere regions of fission yeast.. EMBO J.

[17] Rockmill B, Voelkel-Meiman K, Roeder G. S (2006). Centromere-proximal crossovers are associated with precocious separation of sister chromatids during meiosis in *Saccharomyces cerevisiae*.. Genetics.

[18] Mather K (1939). Crossing over and heterochromatin in the X chromosome of *Drosophila melanogaster*.. Genetics.

[19] Slatis H. M (1955). A reconsideration of the brown-dominant position effect.. Genetics.

[20] Westphal T, Reuter G (2002). Recombinogenic effects of suppressors of position-effect variegation in *Drosophila*.. Genetics.

[21] Yan H, Jin W, Nagaki K, Tian S, Ouyang S (2005). Transcription and histone modifications in the recombination-free region spanning a rice centromere.. Plant Cell.

[22] Carbon J, Clarke L (1984). Structural and functional analysis of a yeast centromere (CEN3).. J Cell Sci.

[23] Furuyama S, Biggins S (2007). Centromere identity is specified by a single centromeric nucleosome in budding yeast.. Proc Natl Acad Sci U S A.

[24] Chen S. Y, Tsubouchi T, Rockmill B, Sandler J. S, Richards D. R (2008). Global analysis of the meiotic crossover landscape.. Dev Cell.

[25] Lambie E. J, Roeder G. S (1988). A yeast centromere acts in cis to inhibit meiotic gene conversion of adjacent sequences.. Cell.

[26] Smith G. P (1976). Evolution of repeated DNA sequences by unequal crossover.. Science.

[27] Schueler M. G, Dunn J. M, Bird C. P, Ross M. T, Viggiano L (2005). Progressive proximal expansion of the primate X chromosome centromere.. Proc Natl Acad Sci U S A.

[28] Ma J, Wing R. A, Bennetzen J. L, Jackson S. A (2007). Plant centromere organization: A dynamic structure with conserved functions.. Trends Genet.

[29] Jaco I, Canela A, Vera E, Blasco M. A (2008). Centromere mitotic recombination in mammalian cells.. J Cell Biol.

[30] Winkler H (1930). Die konversion der gene.

[31] Blitzblau H. G, Bell G. W, Rodriguez J, Bell S. P, Hochwagen A (2007). Mapping of meiotic single-stranded DNA reveals double-stranded-break hotspots near centromeres and telomeres.. Curr Biol.

[32] Buhler C, Borde V, Lichten M (2007). Mapping meiotic single-strand DNA reveals a new landscape of DNA double-strand breaks in *Saccharomyces cerevisiae*.. PLoS Biol.

[33] Symington L. S, Petes T. D (1988). Meiotic recombination within the centromere of a yeast chromosome.. Cell.

[34] Jin W, Melo J. R, Nagaki K, Talbert P. B, Henikoff S (2004). Maize centromeres: Organization and functional adaptation in the genetic background of oat.. Plant Cell.

[35] Zhong C. X, Marshall J. B, Topp C, Mroczek R, Kato A (2002). Centromeric retroelements and satellites interact with maize kinetochore protein CENH3.. Plant Cell.

[36] Wolfgruber T. K, Sharma A, Schneider K. L, Albert P. S, Koo D. H (2009). Maize centromere structure and evolution: Sequence analysis of centromeres 2 and 5 reveals dynamic loci shaped primarily by retrotransposons.. PLoS Genet.

[37] Ranere A. J, Piperno D. R, Holst I, Dickau R, Iriarte J (2009). The cultural and chronological context of early holocene maize and squash domestication in the Central Balsas River Valley, Mexico.. Proc Natl Acad Sci U S A.

[38] Yandeau-Nelson M. D, Zhou Q, Yao H, Xu X, Nikolau B. J (2005). MuDR transposase increases the frequency of meiotic crossovers in the vicinity of a mu insertion in the maize a1 gene.. Genetics.

[39] Brown S. D, Dover G. A (1980). Conservation of segmental variants of satellite DNA of *Mus musculus* in a related species: *Mus spretus*.. Nature.

[40] Henikoff S, Ahmad K, Malik H. S (2001). The centromere paradox: Stable inheritance with rapidly evolving DNA.. Science.

[41] Szostak J. W, Orr-Weaver T. L, Rothstein R. J, Stahl F. W (1983). The double-strand-break repair model for recombination.. Cell.

